# Chemoradiation and consolidation chemotherapy for rectal cancer provides a high rate of organ preservation with a very good long-term oncological outcome: a single-center cohort series

**DOI:** 10.1186/s12957-022-02816-7

**Published:** 2022-11-10

**Authors:** Oktar Asoglu, Alisina Bulut, Vusal Aliyev, Guglielmo Niccolò Piozzi, Koray Guven, Barıs Bakır, Suha Goksel

**Affiliations:** 1 Bogazici Academy for Clinical Sciences, Istanbul, Turkey; 2 Division of Colon and Rectal Surgery, Department of Surgery, Korea University Anam Hospital, Korea University College of Medicine, Seoul, Republic of Korea; 3 Department of Radiology, Acibadem Mehmet Ali Aydınlar University School of Medicine, Istanbul, Turkey; 4 Department of Radiology, Istanbul University Faculty of Medicine, Istanbul, Turkey; 5 Department of Pathology, Maslak Acibadem Hospital, Istanbul, Turkey

**Keywords:** Watch and wait, Non-operative management, Rectal cancer, Clinical complete response, Pathological complete response, Total neoadjuvant chemoradiotherapy

## Abstract

**Aim:**

To report long-term oncological outcomes and organ preservation rate with a chemoradiotherapy-consolidation chemotherapy (CRT-CNCT) treatment for locally advanced rectal cancer (LARC).

**Method:**

Retrospective analysis of prospectively maintained database was performed. Oncological outcomes of mid-low LARC patients (*n*=60) were analyzed after a follow-up of 63 (50–83) months. Patients with clinical complete response (cCR) were treated with the watch-and-wait (WW) protocol. Patients who could not achieve cCR were treated with total mesorectal excision (TME) or local excision (LE).

**Results:**

Thirty-nine (65%) patients who achieved cCR were treated with the WW protocol. TME was performed in 15 (25%) patients and LE was performed in 6 (10%) patients. During the follow-up period, 10 (25.6%) patients in the WW group had regrowth (RG) and 3 (7.7%) had distant metastasis (DM). Five-year overall survival (OS) and disease-free survival (DFS) were 90.1% and 71.6%, respectively, in the WW group. Five-year OS and DFS were 94.9% (95% CI: 88–100%) and 80% (95% CI: 55.2–100%), respectively, in the RG group. For all patients (*n*=60), 5-year TME-free DFS was 57.3% (95% CI: 44.3–70.2%) and organ preservation-adapted DFS was 77.5% (95% CI: 66.4–88.4%). For the WW group (*n*=39), 5-year TME-free DFS was 77.5% (95% CI: 63.2–91.8%) and organ preservation-adapted DFS was 85.0% (95% CI: 72.3–97.8%).

**Conclusion:**

CRT-CNCT provides cCR as high as 2/3 of LARC patients. Regrowths, developed during follow-up, can be successfully salvaged without causing oncological disadvantage if strict surveillance is performed.

## Introduction

Standard treatment of locally advanced rectal cancer (LARC) is neoadjuvant chemoradiotherapy and radical surgery following principles of total mesorectal excision (TME) [1, 2]. However, many patients may suffer rather than benefit from this “one size fits all” strategy. Surgery for LARC is challenging and associated with serious complications as permanent stoma, sepsis following anastomotic leakage, and sexual/urinary dysfunction following nerve injury [3, 4]. LARC surgery is also associated with poor fecal function with high risk of developing low anterior resection syndrome (LARS) in the case of sphincter-preserving resections. Approximately 50–90% of patients undergoing low anterior resection develop LARS symptoms [5], and 5% of these patients will require permanent stoma [6]. LARC surgery carries also a perioperative mortality risk of 1–2% according to the patient’s age and clinical conditions [7, 8]. Adoption of TME, multimodal therapy, and standard use of rectal magnetic resonance imaging (MRI) for clinical staging has rapidly improved the oncological outcomes of LARC [9]. Therefore, in the last two decades, a great interest focused on the quality of life and functional outcomes (fecal, sexual, and urinary) of patients with LARC [10]. In this direction, the development of a watch and wait (WW) strategy has gained rapid interest because it avoids surgery, protecting patients from morbidities and mortality and providing organ preservation. WW is a treatment option for patients with clinical complete response (cCR) after neoadjuvant treatment. cCR rates are reported with a wide range (26.8-78%) according to the neoadjuvant protocol (short-term radiotherapy (RT), long-term chemoradiotherapy (CRT) only, CRT with consolidation or induction chemotherapy) [11]. Additionally, tumor stage (T1-T4), radiotherapy dosage (40-60Gy) and waiting period affect the cCR rates. Chemoradiotherapy-consolidation chemotherapy (CRT-CNCT) and long waiting period increase the cCR rate. Previously, we reported a high rate of cCR (65%) with CRT-CNCT [11]. CRT-CNCT also increases the pathological complete response (pCR) rate. Garcia-Aguilar et al. reported a statistically significant difference in pCR between CRT+TME and CRT+6 cycles FOLFOX+TME group (18 vs 38%, *p*=0.0036) [12].

There are concerns that WW protocol could hinder the oncologic outcomes achievable with neoadjuvant therapy and TME. However, Martens et al. [13] reported that WW protocol is feasible for complete responders and near-complete responders with a 3-year OS rate of 97% and a regrowth rate of 15%. They proved the WW protocol as an oncologically safe option, and that patients with regrowths can also be successfully treated with salvage surgery. Nasir et al. reported that there was no difference in oncological outcomes between patients who underwent surgery following regrowths developed during WW treatment and those who underwent immediate radical surgery because cCR could not be achieved [14].

This study aims to report the median 5-year long-term oncological outcomes of patients treated with WW by obtaining cCR after CRT-CNCT treatment and long waiting period.

## Material and methods

### Study population

This retrospective study evaluated consecutive series of primary LARC (cT3-4, N-any), located within 10cm from anal verge, who were eligible for CRT-CNCT performed between 2015 and 2018. Data were extracted from a prospectively maintained colorectal database. Institutional Review Board approved the study (#2019-02/4). All patients provided informed consent.

Primary aim was to report 5-year oncological outcomes of patients treated with WW protocol. Secondary aim was to report 5-year oncological outcomes of patients with regrowth (RG) during WW and of TME and local excision (LE) group. Inclusion criteria are as follows: (1) cT3-4, N-any rectal cancer; (2) within 10cm from anal verge; and (3) CRT-CNCT protocol. Exclusion criteria are as follows: (1) synchronous colorectal or other primary tumors.

Clinical staging was performed via colonoscopy with biopsy, thoracic/abdominopelvic CT, and pelvic magnetic resonance imaging (MRI). All patients received CRT-CNCT. All patients were treated with image-guided intensity-modulated radiotherapy or volumetric intensity-modulated arc treatment using 6–10 MV photons. Patients received a pelvic radiotherapy dose of 50.4Gy delivered in 28 fractions with concomitant oral capecitabine 825mg/m^2^ twice daily during radiotherapy. After 4 weeks, all patients were re-evaluated by sigmoidoscopy and pelvic MRI. Consolidation chemotherapy with a total neoadjuvant scheme (TNT) was administered to those who achieved a cCR greater than 50%. Until June 2018, six cycles of consolidation chemotherapy were administered comprising bi-weekly FOLFOX administration (oxaliplatin (85 mg/m^2^) and concomitant leucovorin (400 mg/m^2^) for 2 h followed by a bolus injection of 5-fluorouracil (5-FU, 400 mg/m^2^); then, 5-FU (2400 mg/m^2^) was infused over 46h after CRT. After June 2018 [9], the consolidation chemotherapy regimen was changed to oxaliplatin (130mg/m^2^) on day one plus capecitabine (1000mg/m^2^) twice daily on days 1–14, every 3 weeks for eight cycles (11/60 patients, 18.3%). Endoscopy, MRI, and PET/CT were used to evaluate all responses. Sigmoidoscopy, pelvic MRI, and PET/CT were repeated after consolidation chemotherapy completion and the final clinical decision on cCR assessment was made and recorded prospectively.

### Post-chemoradiotherapy evaluation

cCR criteria followed the endoscopic criteria of Habr-Gama et al. [15]. MRI Tumor Regression Grade (TRG) score, as defined by the MERCURY group [16], and additionally high *b*-value (b1000) diffusion-weighted MRI sequences were evaluated and included in the findings by the radiologist. PET/CT images were reviewed for abnormal uptake in the primary tumor, lymph nodes, and distant sites. PET/CT images were compared with primary tumor activity, background activity, and those in all the previous images for each study stage.

### Watch and wait decision

Clinical decisions were taken after the completion of neoadjuvant treatment. Clinical response criteria used for endoscopy, MRI, and PET/CT were previously reported [11]: 1) white scar on endoscopy, 2) TRG1-2, and 3) diffusion negative in MRI, absence of visual abnormal FDG uptake in PET/CT. If findings were not compatible with cCR in any of the aforementioned three evaluation methods, patients were referred to surgery. Any signs of clinical tumor progression during TNT, either by endoscopy or imaging, indicated for surgery.

### Follow-up

Follow-up protocol for WW, TME, and LE groups was previously reported [11].

#### Statistical analysis

Patient characteristics were summarized using basic descriptive statistics. Continuous variables were presented as median (interquartile range, IQR) or mean±standard deviation accordingly, and compared using the Mann-Whitney *U* test. Categorical variables were expressed as proportions and analyzed using chi-square test. Statistical analysis was performed using IBM SPSS Statistics for Windows, version 22.0 (IBM Corp., Armonk, NY, USA). Overall and disease-free survival rates were estimated through the Kaplan-Meier model and compared by log-rank test. Confidence intervals were estimated at 95%, and the significance level was set at *p*=0.05.

## Results

### Clinical findings

**Fig. 1:**
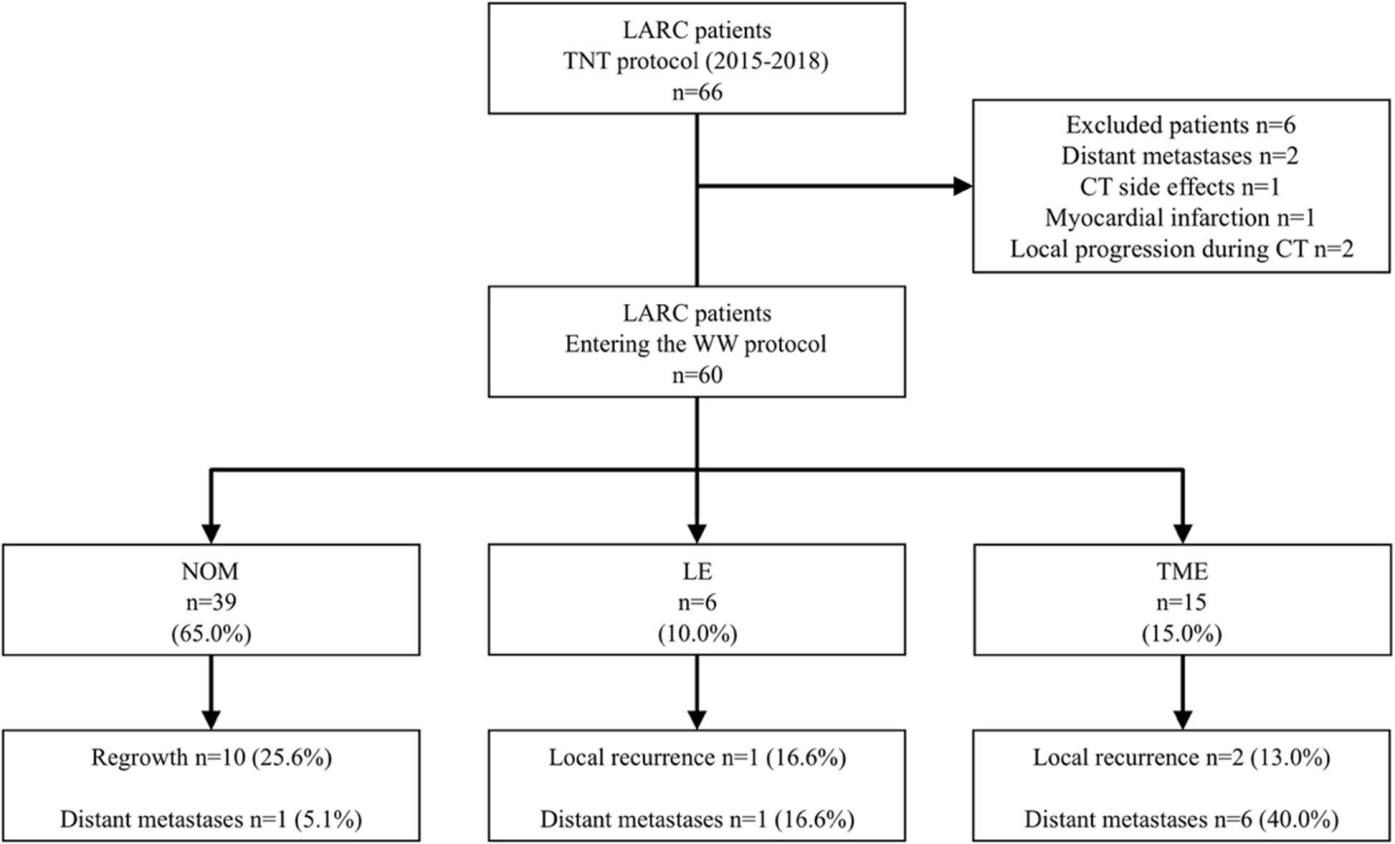
Flowchart showing the patients and treatment protocols

Sixty-six patients with LARC were treated with TNT protocol (CRT-CNCT) between 2015-2018 (Fig. 1). Six patients were excluded from the study [11].

cCR was achieved in 39 (65%) patients after CRT-CNCT; these patients were treated with a WW strategy. TME was applied to 15 (25%) patients who could not achieve cCR. Six (10%) patients formed the LE group. Median follow-up was 63 (50–83) months. Between WW patients, ten (25%) were cT4, and 30 (77%) were N+. Clinical stage III was the most common (82%). Most patients were cCRM+ (54%) and cEMVI negative (68%). Abdominoperineal resection (APR) was initially indicated to 56% of WW patients. Clinical findings are shown in Table 1.

**Table 1:** Tumor stage and treatment parameters

	***n***=60	WW (***n***=39)	TME (***n***=15)	LE (***n***=6)
**cT**				
**T3a**	10 (17%)	8 (20%)	0	2 (33%)
**T3b**	22 (37%)	15 (38%)	6 (40%)	1 (17%)
**T3c**	9 (15%)	6 (15%)	3 (20%)	0
**T3d**	2 (3%)	0	1 (7%)	1 (17%)
**T4**	17 (28%)	10 (25%)	5 (33%)	2 (33%)
**cN+**	49 (82%)	30 (77%)	15 (100%)	4 (67%)
**Clinical stage**				
**II**	11 (18%)	9 (23%)	0	2 (33%)
**III**	49 (82%)	30 (77%)	15 (100%)	4 (66%)
**cCRM+**	36 (60%)	21 (54%)	12 (80%)	3 (50%)
**cEMVI+**	19 (32%)	8 (21%)	9 (60%)	2 (33%)
**Initial APR indication**	31 (52%)	22 (56%)	6 (40%)	3 (50%)

*APR* abdominoperineal resection, *cCRM* clinical circumferential resection margin, *cEMVI* clinical extramural vascular invasion, *LE* local excision, *TME* total mesorectal excision, *WW* watch-and-wait protocol

### Oncological outcomes

#### WW group regrowth rate

Local regrowth was reported in ten (25.6%) patients in the WW group (Table 2). Median time for regrowth was 20 (8–36) months. Local regrowth rate at the first, second, and third years was 7.7, 20.5, and 25.6%, respectively. No further local regrowth was reported after the third year of follow-up. During follow-up, sustained WW rate was 48%. Salvage treatment included five (50%) LE (one with additional brachytherapy) and five (50%) TME (three low anterior resection (LAR) and two APR). One patient, who was submitted to LE (ypT2), developed a local recurrence after 11 months and underwent APR as second salvage surgery (ypT2N0). This patient has no evidence of disease after 59 months from APR. Another patient, who was submitted to LE (ypT3) after 17 months of follow-up, developed a local recurrence after 9 months which was treated with laparoscopic LAR (ypT3N0). After 8 months, a new local recurrence was detected and an APR was performed (ypT3N1). This patient has no evidence of disease after 67 months from APR.

**Table 2:** Surgical and oncological outcomes of patients with local regrowth

n	Clinical stage	Regrowth, months	Salvage surgery	Pathology after salvage	Local recurrence, months	Distant metastases	Follow-up, months	State of patient
1	cT3N+	24	LAR	ypT2N0		No	68	NED
2	cT3N+	24	LE	ypT2		No	68	NED
3	cT3N+	18	LE/APR	ypT2/ypT2N0	11	No	66	NED
4	cT3N+	11	LE	ypT2		No	62	NED
5	cT3N+	10	LAR	ypT2N0		No	59	NED
6	cT3N+	17	LE/LAR/APR	ypT3/ypT3N0/ypT3N1	9, 8	No	74	NED
7	cT3N+	20	LAR	ypT3N1a		No	67	NED
8	cT3N+	36	APR	ypT4N0		No	60	NED
9	cT3N+	8	LE	ypT2		No	64	NED
10	cT3N+	35	APR	ypT3N2		Yes	48	DOD

*APR* abdominoperineal resection, *DOD* dead of disease, *LAR* low anterior resection, *LE* local excision, *NED* no evidence of disease

Only one patient, who could not be followed up for 2 years because of the COVID-19 pandemic, developed a regrowth after 36 months which required an APR. This was the only ypT4.

Only one patient (age 83 years) is dead of disease at follow-up after developing systemic metastases and dying 48 months after salvage surgery (APR). The had a mixt adeno-neuroendocrine cancer (MANEC). This was the only patient in the salvage group (10%) developing distant metastases (DM). All other patients (90%) are still alive with no evidence of disease (NED).

R0 resection was achieved in all patients undergoing salvage surgery. Initial APR indication in salvage patients was 20%; however, following secondary and tertiary recurrences, APR was performed in 40% of cases. Interestingly, 60% of patients (*n*=6) treated with salvage surgery are still alive without permanent colostomy.

Except for the salvage patients, DM were detected in two patients (5.1%): (1) bone metastases after 14 months and (2) lung metastases after 8 months.

**Fig. 2:**
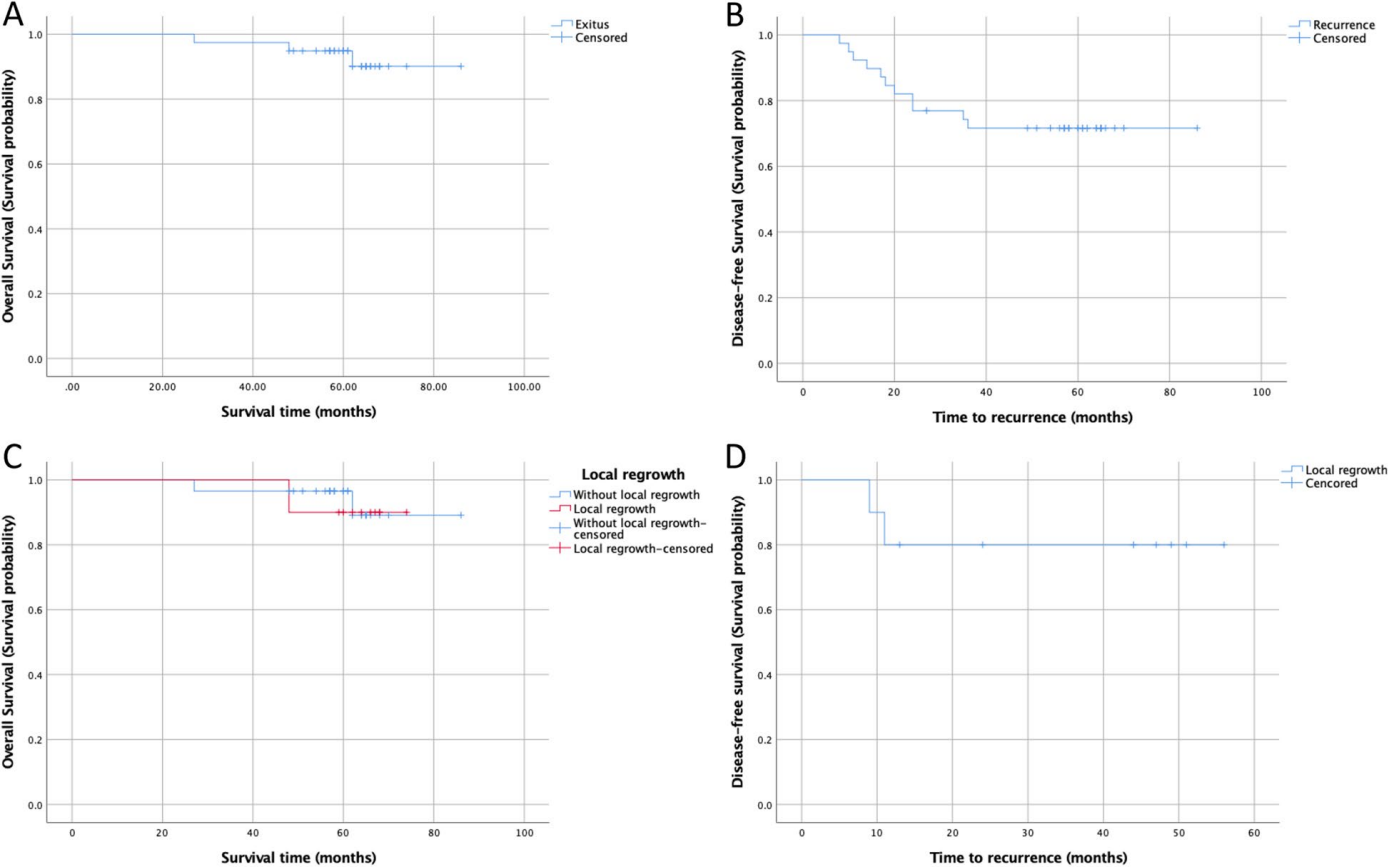
**a** Overall survival curve in patients treated with WW protocol (5-year OS 90.1% (95% CI: 78.9–100.0%). **b** Disease-free survival curve in patients treated with WW protocol (5-year DFS 71.6% (95% CI: 57.4–85.8%). **c** Overall survival curve in patients with non-RG and RG [(5-year OS: 90.1% (95% CI: 78.9–100.0%) and 94.9% (88.0–100.0%) (Log-rank test *p*-value: 0.87)]. **d** Disease-free survival curve in patients with RG (5-year DFS: 80.0% (95% CI: 55.2–100.0%)

Five-year OS and DFS were 90.1% and 71.6%, respectively (Fig. 2a, b). Ten patients who developed regrowth and 29 patients who did not develop regrowth were also evaluated in terms of oncological outcomes. Five-year OS and DFS were 94.9 (95% CI: 88–100%) and 80% (95% CI: 55.2–100%), respectively, in the regrowth group (Fig. 2c, d). Five-year OS and DFS were 90.1 (95% CI: 78.9–100%) and 96.6% (95% CI: 89.9–100%), respectively, in the non-regrowth group (Fig. 2c).

#### TME group

All patients who could not achieve cCR and were unsuitable for LE underwent sphincter-saving surgery. Intersphincteric resection with coloanal anastomosis was performed in seven (46.6%) patients [17]. DM developed in 6 (40%) patients: three in the lung only, two in the lung and liver, and one as a peritoneal implant. Two (13%) patients developed local recurrence. One patient developed a local recurrence after 47 months. The patient refused surgery and preferred chemotherapy-only treatment. The patient later developed liver metastasis after 56 months and is alive at 66 months. Another patient developed local recurrence in the vagina after 9 months of follow-up. She was treated with a transvaginal local excision. She developed lung-liver metastases after 15 months and died at 28 months of follow-up.

**Fig. 3:**
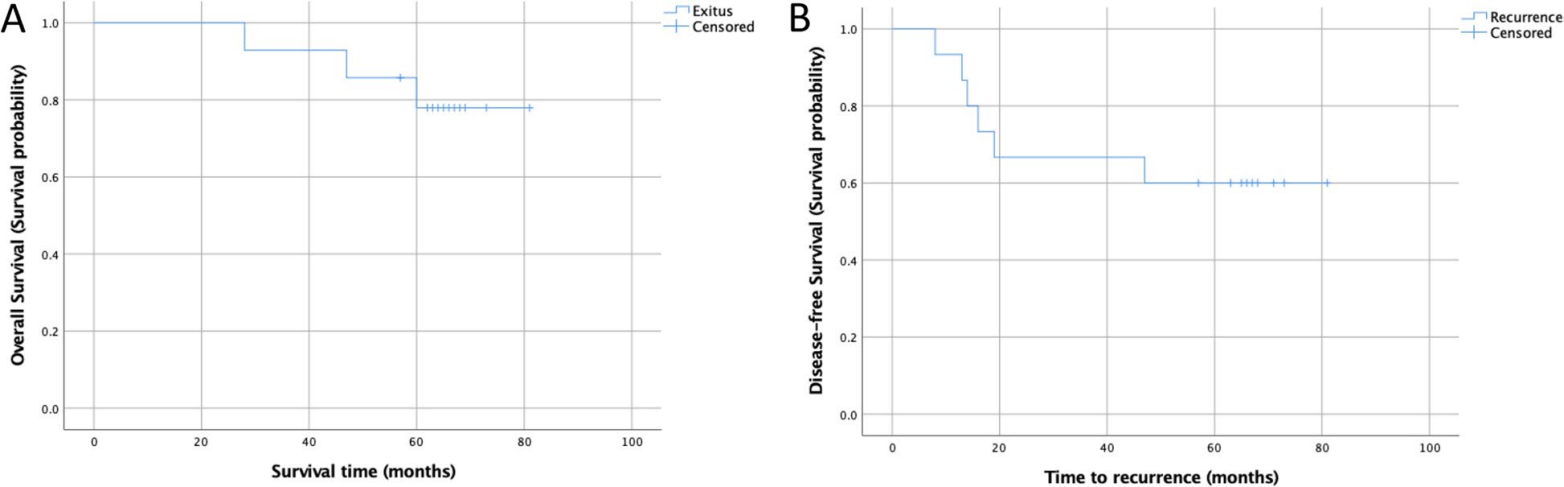
**a** Overall survival curve in patients treated with TME (5-year OS: 78% (95% CI: 55.8–100.0%). **b** Disease-free survival curve in patients with TME (5-year DFS: 60% (95% CI: 35.2–84.8%)

In this group, a total of three patients died at 28, 47, and 60 months due to disease progression. Five-year OS and DFS were 78% (95% CI: 55.8–100%) and 60% (95% CI: 35.2–84.8%), respectively, for the TME group (Fig. 3a, b).

#### LE group

DM was detected in one (16.6%) patient after 23 months, and lung metastasectomy was performed. After 8 months, local recurrence was detected in another patient (16.6%) who then underwent intersphincteric resection (ypT3N1) as a salvage surgery with the initial ypT3 pathology. Five-year OS and DFS were 100% and 66.8%, respectively.

#### Organ preservation outcomes

**Fig. 4:**
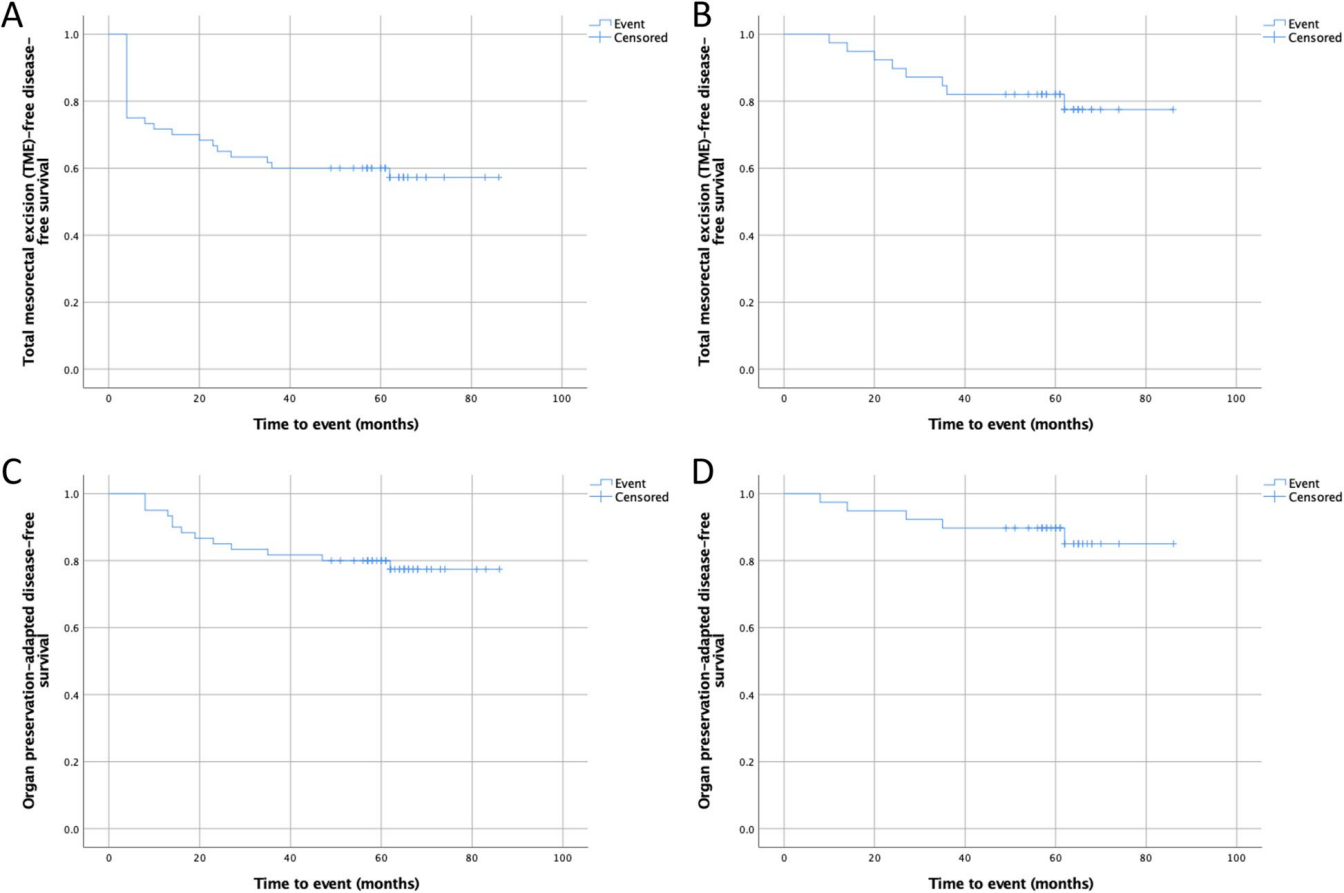
**A** TME-free DFS curve for all patients (*n*=60). **B** TME-free DFS curve for the WW group only (*n*=39). **C** Organ preservation-adapted DFS for all patients (*n*=60). **D** Organ preservation-adapted DFS for the WW group only (*n*=39)

Following the international consensus statement on key outcome measures for organ preservation after chemoradiotherapy in patients with rectal cancer from Fokas et al. [18] the TME-free DFS and organ preservation-adapted DFS were calculated at 5 years for all patients (*n*=60) and for the WW group only (*n*=39) (Fig. 4).

For all patients, TME-free DFS was 57.3% (95% CI: 44.3–70.2%) and the organ preservation-adapted DFS was 77.5% (95% CI: 66.4–88.4%). For the WW group only, TME-free DFS was 77.5% (95% CI: 63.2–91.8%) and the organ preservation-adapted DFS was 85.0% (95% CI: 72.3–97.8%).

### Colostomy-free patients

Six patients underwent APR due to regrowth and LR. Moreover, a loop colostomy was performed to a patient who developed total incontinence after intersphincteric resection. Despite the initial APR indication being 52% in all patients, at the end, 88% of patients were colostomy-free.

## Discussion

WW protocol, by providing organ preservation, is attracting increasing attention for treatment of rectal cancer and more patients are beginning to ask about this alternative treatment [14]. This study shows that WW provides a high rate of organ preservation (62%) with little or no apparent oncological risk.

Median cCR rate described in literature was 65% (range 55–75%) [11, 12, 19–21] which was equivalent to ours (Table 3). Local excision was required in 10% of patients, and a total of 75% of organ preservation was achieved. In the phase 2 OPRA study [12], which had a design similar to ours regarding treatment protocol and waiting period, the cCR rate was 75% in the consolidation arm. This slightly higher cCR rate may be related to differences in sample size, a little longer waiting period, and T1–T2 tumors consisting of 12% of WW patients in the consolidation arm. Tumor and treatment-related factors such as waiting period, clinical MRI findings, adding neoadjuvant CRT with or without chemotherapy affect cCR rates. cCR is closely related to the cT stage [22]. We retrospectively examined the relationship between the cT stages of patients who received cCR and the duration of the cCR in a previous study [22]. In this study, we showed that, after a waiting period of 8–10 weeks, cCR was as high as 78.5% in cT3a tumors, 45% in cT3b, and 17% in cT3–cT4 tumors. This result is crucial as it shows that tumors with early T stage are cleared at a higher rate after shorter waiting periods. In studies with long-course RT, but with a maximum waiting period of 8 weeks after RT, cCR rates ranged 11–19% [23–25]. A review [26], with a majority of studies using standard CRT, reported that the waiting period for reassessment ranged between 3 and 24 weeks and the cCR was 22.4% on average. CRT-CNCT protocol provides higher rates of cCR in comparison with the other protocols.

**Table 3:** Studies using WW protocol after CRT-CNCT

Study	Patients, ***n***	cT stage	Neoadjuvant protocol	CT regimen	RT Dose	^a^Clinical decision, weeks	cCR	^b^RG	Follow-up, months	OS/DFS
Rettig et al. [19]	66	T1: 1 (1.50%) T2: 9 (13.6%) T3: 50 (75.8%) T4: 6 (9.10%)	CRT-CNCT (84%) INCT-CRT (16%)	8 FOLFOX 6 CAPEOX	50.4 Gy	20–22	55%	19%	30	86%/NR
Garcia-Aguilar et al. [12]	158	T1–2: 13 (12%) T3: 82 (77.0%) T4: 11 (10.0%)	CRT-CNCT	8 FOLFOX 5 CAPEOX	50–56 Gy	28.5	75%	19%	36	NR/76%
Habr-Gama et al. [20]	126	T1: 0. (0.00%) T2: 27 (55.0%) T3: 20 (40.8%) T4: 2 (4.20%)	CRT-CNCT	6 cycles 5FU+Leucovorin	54 Gy	10	63%	27%	NR	NR/NR
Habr-Gama et al. [21]	69	T1: 0 (0.00%) T2: 20 (28.6%) T3: 47 (67.1%) T4: 3 (4.30%)	CRT-CNCT	6 cycles 5FU+Leucovorin	50.4 Gy	10	68%	25%	56	90%/72%
Present Study	60	T1: 0 (0.00%) T2: 0 (0.00%) T3: 43 (72.0%) T4: 17 (18.0%)	CRT-CNCT	6 FOLFOX 6 XELOX	50.4 Gy	20–26	65%	25%	63	90.1%/71.6%

*cCR* complete clinical response, *CRT-CNCT* chemoradiotherapy-consolidation chemotherapy, *CT* chemotherapy, *DFS* disease-free survival, *INCT-CRT* induction chemotherapy-chemoradiotherapy, *NR* non reported, *OS* overall survival, *RG* regrowth, *RT* radiotherapy, *WW* watch-and-wait protocol

^a^Time interval end of radiotherapy and reassessment

^b^Ratio of patients with RG to total patients in the WW protocol

Major concerns of WW protocols are regrowth, development of DM, and generally worse oncological outcomes. Regrowth ranges and salvage surgery details in literature are shown in Table 4. Median regrowth rate was 25% (19–31%) [14, 27–32]. Duration of follow-up could be the main reason in different regrowth rates. Median follow-up duration in our study was 63 (50–83) months in the WW group. Ten patients (25.6%) in the WW group had regrowth, and all were salvaged through surgery (LE=5, LAR=3, APR=2).

**Table 4:** Studies on regrowth and salvage surgery

Study	Patients (WW group), ***n***	Regrowth rate	Salvage rate	Salvage type	Pathology of LE group	Follow-up, months	OS/DFS (patients with regrowth)
Habr-Gama et al. [27]	90	28 (31%)	93%	LAR: 7 (26%) APR: 11 (42%) LE: 7 (26%) BRACYTX: 1 (1%)	T1: 2 (28.5%) T2: 5 (71.5%)	60	88%/78%
van der Valk et al. [28]	880	213 (24%)	69%	TME: 78% LE: 31%	NR	40	75.4%/NR
Smith et al. [29]	113	22 (19%)	100%	LAR: 9 (41%) APR: 10 (45%) LE: 2 (0.9%) Perineal Resection: 1 (0.4%)	T1: 1 NR: 1	43	NR/NR
Nasir et al. [14]	78	23 (29%)	100%	LAR: 10 (43.5%) APR: 9 (39.1%) LE: 4 (17.4%)	T0: 1 (75%) T2: 3 (25%)	36	NR/NR
Fernandez et al. [30]	257	73 (28%)	94%	LAR: 19 (27%) APR: 24 (34%) LE: 22 (31%) BRACYTX: 1 (0.1%) NR: 3 (0.4%)	NR	40	NR/NR
van der Sande et al. [31]	385	89 (23%)	94%	LAR: 34 (40%) APR: 24 (27%) LE: 26 (31%)	T0: 5 (19%) T1: 4 (15%) T2: 15 (58%) T3: 1 (4%) Tx: 1 (4%)	28	98.4%/90.3%
Geubels et al. [32]	591	175 (29%)	94%	TME: 89 (50%) LE: 77 (44%)	T0: 28 (36%) T1: 11 (14%) T2: 32 (41%) T3: 6 (0.7%)	53	96%/NR
Present study	39	10 (25%)	100%	LAR: 3 (30%) APR: 2 (20%) LE: 5 (50%)	T2: 4 (80%) T3: 1 (20%)	63	95%/80%

*APR* abdominoperineal resection, *BRACYTX* brachytherapy, *LAR* low anterior resection, *LE* local excision, *NR* non reported, *WW* watch-and-wait protocol

DM developed in three (7.7%) patients, one of whom was in the regrowth group. This very low result was similar to the 5-year DM rate of 6.5% reported by the meta-analysis from Socha et al. [33] confirming the low risk of developing DM in the WW protocol. Regrowth rate in the consolidation arm of the OPRA trial [12] was reported as 27%, comparable to ours. Although all regrowths were successfully treated with salvage surgery in many studies, including ours, it should be reported that 2–3% of regrowths cannot be salvaged [23]. As shown in Table 4, LE or TME is preferred in patients requiring salvage surgery. The reason why a high rate of salvage surgery can be performed and LE can be used frequently as a salvage surgery method is that regrowth can be detected at an early stage with a strict follow-up protocol, as performed in the present study.

As a salvage surgical technique, we applied LE with a high rate (50%). The low number of patients in the salvage group is the reason for this high rate. The literature reports the application of LE ranging between 1 and 44% [14, 27–32]. Fernandez et al. applying 32% of LE reported 14% rate of local recurrence after LE [30]. In another report [31], LE was performed in 30% of patients with a local recurrence of 8%. Geubels et al. performed 44% of LE and reported a 19% rate of local recurrence [32]. In the present study, recurrence requiring radical surgery occurred in two (40%) of five patients who underwent LE in patients with regrowth. Between patients who developed regrowths, four patients underwent APR. APR was performed only in 10% of patients in the WW group despite the initial indication was 56%.

In the present study, no newly detected regrowths were reported after the third year of follow-up. Fernandez et al. emphasized that the intensity of active surveillance can be reduced after the first 3 years [34]. Van der Valk et al. [28] revealed that 88% of local regrowths occurred in the first 2 years, and 97% were detected as luminal. Although APR was performed due to recurrences in the LE group, 60% of the patients could continue their lives without APR. In the consolidation arm of the OPRA trial [17], TME was recommended for all local regrowths due to the study design. TME was performed in 81% of those with local regrowth, and LE was performed in 9% of patients. Although patients who underwent LE are considered to have TME due to the intention-to-treat, actual organ preservation rate in these patients was 60%. Although radical surgery can be successfully performed for local recurrences, risk of recurrence after LE is high. LE may be considered as an option in patients who are unsuitable for radical surgery due to comorbidities or do not want to live with a permanent stoma.

In the present study, the salvaged treated patients’ 5-year OS and DFS were 94.9% and 80%, respectively (Figs. 3 and 4). In the OPRA study [12], the DFS rates of patients who underwent TME at the initial evaluation and those who underwent TME after local regrowth were similar. According to Van der Valk et al.’s report [28], 3-year rate of DM was 8.1%. While the 5-year OS of those with local regrowth was 75.4%, the DM rate of these patients was 17.8%. Maas et al. [35] reported that 5-year OS of patients with non-pCR was 76.5%, and DM rate of these patients was 22.7% [28]. Five-year OS rates of patients with sustained cCR without local regrowth were similar to those of patients with pCR in the Maas et al. study (87.9% vs 87.6%). These data reveal that regrowths do not cause a disadvantage in terms of oncological outcomes when detected and treated at an early stage.

Five-year OS was 90.1%, and DFS was 71.6% in our WW group. In the consolidation arm of the OPRA study [12], OS was 80% and DFS was 75%. Habr-Gama et al. reported a 3-year OS of 90% and 3-year DFS of 72% in patients treated with WW [21]. The study also included patients who underwent TME after TNT, the 3-year OS was reported as 86%. Our long-term oncological outcomes are compatible with the literature. In this study, patients who could not achieve cCR constituted the TME group. In this group, who responded poorly to neoadjuvant therapy, higher rates of local recurrence and DM were related to tumor features. Five-year OS and DFS were 78% (95% CI: 55.8–100%) and 60% (95% CI: 35.2–84.8%), respectively, in the TME group. The lower rates of OS and DFS compared to the WW group could be related to poorer response to neoadjuvant therapy in this group.

This study also reported the newly described organ preservation outcomes following the international consensus statement on key outcome measures for organ preservation after chemoradiotherapy in patients with rectal cancer [18]. Interestingly, for the WW group only, TME-free DFS and organ preservation-adapted DFS were very high (77.5% and 85.0%, respectively) which supports the importance in pursuing a WW protocol with consequent organ preservation. However, since our report is one of the first to report these outcome measurements, further studies are needed to compare our results with the literature.

This study has limitations. First, it is a retrospective study potentially suffering from patient selection bias. Second, the series is relatively small. However, it has several strengths. First, it was designed as a phase 2 OPRA study as a treatment and follow-up protocol. Second, it provides detailed long-term oncological outcomes for all groups. Third, it is one of the first studies presenting the newly described organ preservation outcomes.

## Conclusion

Long course CRT followed by administration of TNT with consolidation chemotherapy and a long waiting period provides organ preservation in approximately 2/3 of LARC patients. There is a risk of developing regrowths in approximately 25% of patients in the first 3 years of follow-up with the WW protocol. As a result, approximately half of the patients can be treated with the WW protocol, which provides organ preservation and prevents surgical morbidity and mortality. While the WW approach provides these benefits, it does not cause a disadvantage in oncological outcomes. Local recurrences and distant metastases occurring in patients treated with WW are at acceptable rates and can be successfully treated with salvage therapy.

## Data Availability

The datasets used or analyzed during the current study are available from the corresponding author upon reasonable request.
